# On the frontlines: An exploratory analysis of unequal exposure to air pollution and COVID-19 in the United States

**DOI:** 10.1186/s12939-022-01705-5

**Published:** 2022-08-04

**Authors:** Daniel Vila, Thomas K. J. McDermott

**Affiliations:** Centre for Economic Research on Inclusivity and Sustainability (CERIS) J.E. Cairnes School of Business and Economics, National University of Ireland Galway, Galway, Ireland

**Keywords:** COVID-19, Air quality, Race, Inequality

## Abstract

Recent literature has suggested a link between poor air quality and worse COVID-19 outcomes. In the United States, this link is particularly noteworthy because of residential sorting along ethnic lines within the US population; minorities are disproportionately exposed to health hazards, including air pollution. The impacts of the COVID-19 pandemic have also been disproportionately concentrated amongst minorities. We explore the association between air quality and COVID-19 outcomes, using county level data for the United States from the first wave of the pandemic in 2020, and test whether exposure to more polluted air can account for some of the observed disparities in COVID-19 outcomes among minorities.

## Introduction

During the first wave of the COVID-19 outbreak in the United States (US), Black and Hispanic people had a higher COVID-19 death rate. Both academic literature [1] and journalistic reporting [2] claims that this can be explained at least partially by a higher prevalence of underlying health conditions (such as diabetes, obesity, and asthma) in these populations. However, these underlying conditions are themselves often a result of differences in exposure to environmental hazards. Non-white populations, especially Black populations, are typically exposed to higher levels of air pollution in the US [3]. According to the American Lung Association, certain communities are also disproportionately exposed to toxic and hazardous waste. These communities most often reside in urban settings, have low socioeconomic status, and include a large proportion of ethnic minorities [4]. This is relevant to an analysis of COVID-19 because of the airborne and respiratory nature of the virus. Indeed, previous studies have demonstrated a link between local air pollution and COVID-19 outcomes [5–8].

Other recent academic literature highlights that COVID-19 has disproportionately impacted racial and ethnic minorities and socioeconomically disadvantaged communities in the US [9–12]. For example, using census tract level data from the city of Chicago, Pierce et al. [9] conclude that communities with larger populations of Non- Hispanic Black and Hispanic individuals experienced not just higher mortality, but also markedly higher loss of potential life due to COVID-19. Historical discrimination in housing policies, and residential sorting along ethnic lines, has resulted in particular groups experiencing long-standing neighborhood disadvantage that includes a number of aspects relevant to COVID-19 outcomes, such as crowded housing, food insecurity, employment instability, higher rates of uninsured residents, and increased reliance on crowded public transit [9, 12, 13].

According to [14], lockdowns in response to COVID-19 actually led to widespread but non-uniform reductions of nitrogen dioxide (NO_2_) and carbon monoxide (CO) consistent with the lower transportation and utility demands that dominate NO_2_ and CO emissions, especially in major urban areas. While there are certainly public health benefits associated with these reductions in local air pollution, the effects associated with air pollution exposure are chronic in nature. As such, short term reductions in the concentrations of local NO_2_ and CO levels do not negate the chronic disproportionate exposure that ethnic minorities in the US have faced for decades. For this reason the scope of this article focuses on long-term exposure to air pollution. We acknowledge that the effects of long-term exposure to pollutants on COVID-19 outcomes may be different from short-term exposures. However, Kerr et al. [15] highlight that prior to the pandemic, satellite-observed NO_2_ levels in the least white census tracts of the United States were nearly triple the NO_2_ levels in the most white tracts. Although the largest reductions occurred in marginalized areas, the effect of lockdowns on racial, ethnic, and socioeconomic NO_2_ disparities was mixed and, for many cities, nonsignificant. For example, the least white tracts still experienced around 1.5 times higher NO_2_ levels during the lockdowns than the whitest tracts experienced prior to the pandemic. Meaning that even though lockdowns may have improved air quality, historical populations who were disproportionately exposed to air pollution are still disproportionately affected even taking the improvements into consideration. This is further highlighted in recent work by [16] where the author performed a bi-variate statistical analysis on the convergence of COVID-19 and air pollution risk burdens in the U.S. They concluded that non-Hispanic Blacks, socioeconomically deprived residents, people with disabilities, and those without health insurance are significantly over represented in this high-high category of counties (high COVID-19 prevalence and high hazardous air pollutant (HAP) respiratory risks), when compared to the rest of the continental U.S. The largest relative disparities were observed for the percentages of non-Hispanic Black population, adults without high school education, and people in poverty. White and older (age 65 or more) residents, in contrast, are significantly over represented in counties with both low COVID-19 prevalence and low HAP respiratory risks.

The relationship between place and health outcomes is hardly a new finding; there is a long-standing literature in geography and social epidemiology on the estimation and interpretation of place effects [17, 18]. While place is certainly an important factor in discussions on health equity, because of past housing discrimination, in the US it is impossible to completely disentangle race and place. The COVID-19 pandemic has brought this discussion to the forefront of health and hospital policy. According to [19], as of 2017, people of color were concentrated in the lowest wage healthcare positions. In the context of the COVID-19 crisis, many of these low-income workers struggled to find or afford child-care, saw job losses in their families, and were furloughed or experienced reduced hours at hospitals across the country. These same employee groups also contracted COVID-19 at higher rates compared to those in higher income brackets; at Brigham and Women’s Hospital (Boston, USA) some of the lower paid employee groups like environmental and food services were testing positive for COVID-19 at up to 10 times the rates of higher wage frontline workers such as physicians and nurses [19].

Our research adds to this emerging discussion on structural inequalities and COVID-19, by exploring the relationship between COVID-19 outcomes, air quality, and race directly. Using county level data for the US, we specifically examine whether or not higher levels of air pollution are associated with worse COVID-19 outcomes in the US, and if this can account for worse COVID-19 outcomes among minorities.

## Data

We construct a dataset at the county level for the continental US, combining information on COVID-19 outcomes, socio-demographic data, air pollution and workplace attendance for some 2,757 counties, for the period 01 January 2020 to 10 June 2020, i.e. the first wave of the pandemic.

Data on COVID-19 outcomes including deaths and confirmed cases, as well as socio-demographic data, all observed at the county level, are from the Johns Hopkins University Center for Systems Science and Engineering dataset (JHU CSSE) [20]. The mean number of cases per county over this period was 600, with a max of 80,204 in Cook County, Illinois. The mean number of deaths per county was 31, with a max of 3,780 also in Cook County, Illinois.^1^

**Table 1:** Summary statistics for the main variables used

	Mean	Std. Dev.	Min.	Max.
Deaths	31.40261	165.8946	0	3780
Cases	599.9804	2909.401	0	80,204
Mean Workplace Attendance	-16.0958	5.4417	-58.33333	-3.614035
Black pop. as percent of total	9.579032	14.58247	0	87.41228
Hispanic pop. as percent of total	9.290927	13.71558	0	99.06877
Percentage of pop. below fed. poverty line	15.69659	6.216326	2.3	55.1
2019 NO2 Concentration	1.21e-10	1	-1.747016	7.288707
Percentage of pop. over age of 65	14.65527	7.685659	0	55.59633
Natural log of population density	3.176304	1.50806	-3.148377	10.23119
Respiratory illness mortality rate per 100,000 (2018)	112.3384	58.60863	0	574.4
Population	114,264	344,241.1	1605	10,100,000
Cases/pop.	.0035391	.0063653	0	.1456179
ICU beds per capita	.0002558	.0002703	0	.0041118

For air pollution, we obtain data on NO_2_ concentrations from NASA’s Goddard Earth Sciences Data and Information Services Center (GES DISC). NO_2_ is primarily produced by burning fuel. As such, local sources of NO_2_ pollution include power plants, cars, trucks, buses, and other combustion engines. According to the US EPA, breathing air with a high concentration of NO_2_ can irritate airways in the human respiratory system. Such exposures over short periods can aggravate respiratory diseases, particularly asthma, leading to respiratory symptoms (such as coughing, wheezing or difficulty breathing), hospital admissions and visits to emergency rooms. Longer exposure to elevated concentrations of NO_2_ may contribute to the development of asthma and potentially increase susceptibility to respiratory infections [21]. We take the average NO_2_ concentration over the period January-June for 2019, as a measure of baseline (pre-pandemic) exposure to air pollution. We then merge this data with county boundary shapefiles using GIS software to obtain county level estimates for NO_2_ concentrations. For ease of interpretation we normalise the NO_2_ concentrations to have mean 0 and standard deviation of 1.

Finally, data on workplace attendance is based on Google’s COVID-19 Community Mobility Reports [22]. These reports contain daily observations of workplace attendance at the county level, which we aggregate to obtain a measure of the average change over the period, relative to the same period in 2019. Not surprisingly, all counties experienced a decline in workplace mobility, with an average 16% decline relative to the pre-COVID-19 baseline.

**Fig. 1:**
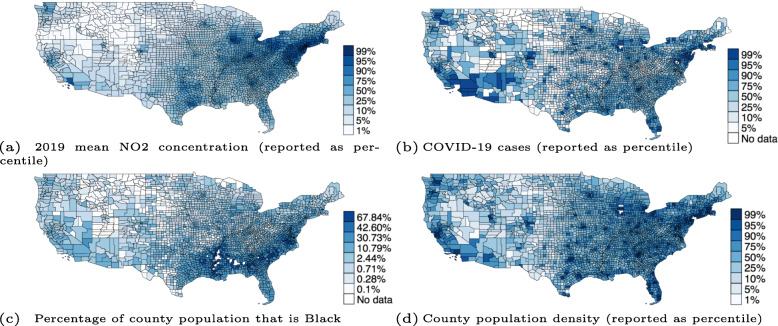
Geographic distribution of NO_2_ concentration, COVID-19 cases, percentage of county population that is Black, and population density at the county level

Figure 1 displays maps at the county level for NO_2_ concentration, COVID-19 cases, percentage of county population that is Black, and population density, in our sample. Evident from these maps is the geographic concentration of NO_2_ concentrations and cases, both of which are associated primarily with high density urban areas.

## Methods

Our aim is to investigate if counties with a higher share of Black or Hispanic population experienced worse COVID-19 outcomes, and the extent to which these outcomes may be related to exposure to poor air quality. We start by estimating models of the number of COVID-19 related deaths as the outcome variable. Given the over-dispersion of the outcome variable (county level deaths has a mean of 31 and a standard deviation of 166 in our sample period) and the relatively large number of zeros (nearly 1200 counties had zero Covid deaths), zero inflated negative binomial (ZINB) regressions were used to estimate these models. The ZINB model is a combination of a binary model of the zero distribution, and a count model of the non-zero data, allowing for the possibility that there are distinct processes in the data leading to zeros and non-zeros in the outcome variable [23]. In our case, this corresponds to counties that experienced zero Covid deaths during our sample period. Many of these counties also experience very few or even no Covid cases over the period.

In addition to mortality outcomes, we are also interested in examining exposure to the virus, and how this has varied across counties in our data. To do so, we estimate models with cases per 1,000 of population as the outcome, using ordinary least squares regression. Finally, we also examine differences in rates of attendance at work across counties, again using ordinary least squares regressions with workplace mobility as the outcome of interest.

## Results

The results of our regression analysis are presented in Table 2.^2^ In the first column, we see that controlling for the number of confirmed cases, the number of deaths from COVID-19 was higher in counties with higher shares of Black or Hispanic population, as well as in counties with higher rates of poverty. In the second column, where we include NO_2_ concentration, we see that the number of deaths from COVID-19 is also higher in counties with worse air quality. However, the inclusion of the NO_2_ variable does not appear to reduce the size or precision of the estimated coefficients on the three socio-demographic variables. Contrary to our initial hypothesis, this suggests that air quality is not the primary mechanism behind worse Covid outcomes for minority groups.

**Table 2:** Summary of key findings from exploratory regression analysis

	(1)	(2)	(3)	(4)	(5)
	ZINB	ZINB	OLS	OLS	OLS
	Deaths	Deaths	Cases/pop.	Workplace attd.	Workplace attd.
Black pop. as percent of total	0.348 ^∗∗∗^	0.393 ^∗∗∗^	0.0725 ^∗∗^	0.0494 ^∗∗∗^	0.0825 ^∗∗∗^
	(0.0839)	(0.107)	(0.0294)	(0.0171)	(0.0247)
Hispanic pop. as percent of total	0.883 ^∗∗∗^	1.223 ^∗∗∗^	0.0429	-0.0110	-0.0111
	(0.144)	(0.220)	(0.0304)	(0.0135)	(0.0132)
Percentage of pop. below fed. poverty line	0.901 ^∗∗∗^	1.430 ^∗∗∗^	-0.0397	0.191 ^∗∗∗^	0.197 ^∗∗∗^
	(0.240)	(0.356)	(0.0654)	(0.0462)	(0.0455)
2019 NO2 Concentration		11.21 ^∗∗∗^	2.759 ^∗∗∗^		
		(2.364)	(0.455)		
Cases/pop.				-0.0654 ^∗∗^	0.000755
				(0.0323)	(0.0480)
Cases/pop. ^∗^ Black pop. as percent of total					-0.00516 ^∗∗^
					(0.00225)
Constant			4.636 ^∗∗^	-12.92 ^∗∗∗^	-13.28 ^∗∗∗^
			(1.841)	(0.911)	(0.928)
Observations	2,756	2,741	2,741	2,724	2,724
R-squared			0.450	0.503	0.509

Notes:

attd.: attendance

Standard errors in parentheses.

All regressions include (log) population density as an additional control.

Columns (1) and (2) include controls for the percent of county population over age 65

and the number of COVID-19 cases.

Cases/pop. is the number of cases per 1,000 of county population.

Regressions 3, 4, and 5 are also analytically weighted by the total pop. and clustered by state.

^∗∗∗^*p*< 0.01,^∗∗^*p*< 0.05,^∗^*p*< 0.1

**Table 3:** Results from the first stage inflation model for the mortality outcomes

	(1)	(2)
	(ZINB)	(ZINB)
	Deaths	Deaths
Marginal Effects		
Black pop. as percent of total	0.348 ^∗∗∗^	0.393 ^∗∗∗^
	(0.0839)	(0.107)
Hispanic pop. as percent of total	0.883 ^∗∗∗^	1.223 ^∗∗∗^
	(0.144)	(0.220)
Percentage of pop. below fed. poverty line	0.901 ^∗∗∗^	1.430 ^∗∗∗^
	(0.240)	(0.356)
2019 NO2 Concentration		11.21 ^∗∗∗^
		(2.364)
Inflation Model (Cases)	-0.0594 ^∗∗∗^	-0.0584 ^∗∗∗^
	(0.007)	(0.007)
Observations	2,756	2,741
R-squared		

Notes:

Standard errors in parentheses.

Additional controls: (Log) of population density and percent of pop. over 65.

^∗∗∗^*p*< 0.01,^∗∗^*p*< 0.05,^∗^*p*< 0.1

In each of these regressions we also control for population density and the share of county population over the age of 65. In additional specifications not reported, we included a range of further controls, including the number of intensive care unit beds per capita, rate of mortality from respiratory disease (2018), and the percentage of county population without health insurance, or who smoke. In each case the results on the main variables of interest remain qualitatively unchanged.

A higher count of COVID-19 deaths could reflect greater exposure to the virus, or higher mortality conditional on contracting the virus, or some combination of these. However, we found no statistically significant differences in COVID-19 mortality, conditional on exposure to the virus, for counties with higher shares of Black or Hispanic populations, or higher rates of poverty.^3^ This suggests that whatever explains the significant differences in the count of COVID-19 deaths by county level socio-demographic characteristics, is driven by differences in exposure to the virus, as opposed to differences in outcomes conditional on exposure.

In Column (3) of Table 1, where the outcome is confirmed cases per 1,000 of population, the results show that counties with a higher share of Black population experienced a higher incidence of COVID-19 cases (relative to population), controlling for population density. We also find that the incidence of COVID-19 is higher in counties with worse air pollution.

Finally, in Columns (4) and (5), we test for differences in attendance at work. As expected, in Column (4) we find a negative association with COVID-19 cases, suggesting that where the virus is more prevalent, people engage in avoidance behaviour in the form of less attendance at work. We also find that in counties with a higher share of Black population, attendance at work was significantly higher during the first wave. However, in Column (5) the negative coefficient on the interaction between cases and share of Black population indicates that when infection rates are high, avoidance behaviour in counties with high share of Black population is stronger than average.

## Discussion

Existing literature has demonstrated a link between air quality and COVID-19 mortality, including in the context of the US [5]. Minority, and particularly Black, populations in the US are typically exposed to worse air quality. The effects of COVID-19 have also been disproportionately concentrated amongst minorities.

In our analysis, we explore the association between air quality and COVID-19 outcomes, using county level data for the United States from the first wave of the pandemic in 2020, and test whether exposure to more polluted air can account for some of the observed disparities in COVID-19 outcomes among minorities.

Our findings show that counties with worse air quality have more Covid cases, and higher mortality. We also find that counties with a higher share of Black population experienced both higher cases and higher mortality from COVID-19 during the first wave. However, these two effects – minorities and air quality – appear to operate independently; that is, at least at the county level, bad air quality appears not to be the primary mechanism behind worse COVID-19 outcomes for minority groups.

Further analysis suggests that counties with a higher share of Black population experienced a higher incidence of the disease, but not necessarily worse outcomes conditional on exposure. This led us to explore differences in exposure. In general people try to avoid the disease, and this is evident in our data – attendance at work is lower in counties with a higher incidence of disease. But we also found that attendance at work is higher for counties with higher minority population share. This could be one of the potential mechanisms behind higher exposure to the disease amongst ethnic minorities.

If people generally prefer to avoid risks, higher exposure to the disease presumably represents some form of constrained choice – for example related to observed differences in the capacity to work from home during the pandemic (see e.g. [24]) – or incomplete information about the risk. However, our finding that avoidance behaviour (reduced attendance at work in response to a high incidence of the disease) is if anything stronger in counties with high minority share, suggests that higher exposure among minorities is not a result of lower awareness or concern about the disease among these populations.

Confounding factors associated with air pollution and health outcomes could distort the relationship between air pollution and COVID-19 explored in this paper. Potential examples include the stage of lockdown and population mobility. Another important limitation to our analysis is potential under reporting of COVID-19 outcomes. Individuals who contracted the virus but were never tested would not be included in the data. In particular, if individuals with lower socio-economic status and living in more polluted areas have less access to testing, it may be that our estimates understate the true relationship between pollution and cases. Finally, the spatial resolution of using county level data may miss more local neighborhood level effects. In particular, identifying the full causal relationships between these inter-related and overlapping factors would require higher resolution data on COVID-19 outcomes, ideally at a neighborhood level. This we leave to future research.

## Data Availability

The datasets analysed during the current study are available from: The COVID-19 Data Repository by the Center for Systems Science and Engineering (CSSE) at Johns Hopkins University, JHU CSSE Github [20]. The Google Community Mobility Reports, Google Mobility [22].

## Footnotes

1 Full summary statistics for the main variables used in our analysis are included in Table 1.

2 Table 2 presents a summary of key findings from our exploratory regression analysis. Results from the first stage “inflation model” for the mortality outcomes are included in Table 3.

3 These results not reported, but available on request.

## References

[1] Millett GA, Jones AT, Benkeser D, Baral S, Mercer L, Beyrer C, Honermann B, Lankiewicz E, Mena L, Crowley JS (2020). Assessing differential impacts of covid-19 on black communities. Ann Epidemiol.

[2] Oppel RA, Gebeloff R, Lai KR, Wright W, Smith M. The fullest look yet at the racial inequity of coronavirus. N Y Times. 2020;5. https://www.nytimes.com/interactive/2020/07/05/us/coronavirus-latinos-african-americans-cdc-data.html. Accessed 15 May 2021.

[3] American Lung Association. Disparities in the Impact of Air Pollution. 2020. https://www.lung.org/clean-air/outdoors/who-is-at-risk/disparities. Accessed 31 May 2021.

[4] American Lung Association (2001). Urban air pollution and health inequities: a workshop report. Environ Health Perspect.

[5] Wu X, Nethery RC, Sabath BM, Braun D, Dominici F. Exposure to air pollution and covid-19 mortality in the united states. MedRxiv. 2020. 10.1101/2020.04.05.20054502. 10.1126/sciadv.abd4049 PMC7673673 33148655

[6] Cole M, Ozgen C, Strobl E (2020). Air pollution exposure and Covid-19 in Dutch municipalities. Environ Res Econ.

[7] Travaglio M, Yu Y, Popovic R, Leal NS, Martins LM. Links between air pollution and covid-19 in England. medRxiv. 2020. 10.1101/2020.04.16.20067405. 10.1016/j.envpol.2020.115859 PMC7571423 33120349

[8] Conticini E, Frediani B, Caro D (2020). Can atmospheric pollution be considered a co-factor in extremely high level of sars-cov-2 lethality in northern italy?. Environ Pollut.

[9] Pierce JB, Harrington K, McCabe ME, Petito LC, Kershaw KN, Pool LR, Allen NB, Khan SS (2021). Racial/ethnic minority and neighborhood disadvantage leads to disproportionate mortality burden and years of potential life lost due to covid-19 in chicago, illinois. Health place.

[10] Wadhera RK, Wadhera P, Gaba P, Figueroa JF, Maddox KEJ, Yeh RW, Shen C (2020). Variation in covid-19 hospitalizations and deaths across new york city boroughs. JAMA.

[11] Price-Haywood EG, Burton J, Fort D, Seoane L (2020). Hospitalization and mortality among black patients and white patients with covid-19. N Engl J Med.

[12] Berkowitz RL, Gao X, Michaels EK, Mujahid MS (2020). Structurally vulnerable neighbourhood environments and racial/ethnic covid-19 inequities. Cities Health.

[13] Egede LE, Walker RJ (2020). Structural racism, social risk factors, and covid-19—a dangerous convergence for black americans. N Engl J Med.

[14] Chen L-WA, Chien L-C, Li Y, Lin G (2020). Nonuniform impacts of covid-19 lockdown on air quality over the united states. Sci Total Environ.

[15] Kerr GH, Goldberg DL, Anenberg SC (2021). Covid-19 pandemic reveals persistent disparities in nitrogen dioxide pollution. Proc Natl Acad Sci.

[16] Chakraborty J (2021). Convergence of covid-19 and chronic air pollution risks: Racial/ethnic and socioeconomic inequities in the us. Environ Res.

[17] Mclafferty S (2019). Place and quantitative methods: Critical directions in quantitative approaches to health and place. Health Place.

[18] Couillard BK, Foote CL, Gandhi K, Meara E, Skinner J (2021). Rising geographic disparities in us mortality. J Econ Perspect.

[19] Sivashanker K, Couillard C, Goldsmith J, Walker N, Eappen S (2020). Addressing the caste system in us healthcare in the era of covid-19. Int J Equity Health.

[20] Dong E, Du H, Gardner L (2020). An interactive web-based dashboard to track covid-19 in real time. Lancet Infect Dis.

[21] Moshammer H, Poteser M, Kundi M, Lemmerer K, Weitensfelder L, Wallner P, Hutter H-P (2020). Nitrogen-dioxide remains a valid air quality indicator. Int J Env Res Public Health.

[22] Google LLC. Google COVID-19 Community Mobility Reports. 2020. https://www.google.com/covid19/mobility/. Accessed 3 June 2021.

[23] Silva JMS, Tenreyro S, Windmeijer F (2015). Testing competing models for non-negative data with many zeros. J Econ Methods.

[24] Bloom N, Davis SJ, Zhestkova Y (2021). Covid-19 shifted patent applications toward technologies that support working from home. AEA Papers and Proceedings, vol. 111.

